# Novel ‘*Candidatus* Liberibacter’ species identified in the Australian eggplant psyllid, *Acizzia solanicola*

**DOI:** 10.1111/1751-7915.12707

**Published:** 2017-04-07

**Authors:** Jacqueline Morris, Jason Shiller, Rachel Mann, Grant Smith, Alan Yen, Brendan Rodoni

**Affiliations:** ^1^ Plant Biosecurity Cooperative Research CentreLPO Box 5012BruceAustralian Capital Territory2617Australia; ^2^ La Trobe UniversityAgriBio5 Ring RoadBundooraVictoria3083Australia; ^3^ Agriculture VictoriaAgriBio5 Ring RoadBundooraVictoria3083Australia; ^4^ INRA/Université d'Angers – IRHS Batiment C42 rue Georges MorelBeaucouzé49071France; ^5^ Plant & Food Research LincolGerald StLincoln7608New Zealand; ^6^ Better Border BiosecurityLincoln7608New Zealand

## Abstract

A novel candidate species of the liberibacter genus, ‘*Candidatus* Liberibacter brunswickensis’ (CLbr), was identified in the Australian eggplant psyllid, *Acizzia solanicola*. This is the first discovery of a species belonging to the liberibacter genus in Australia and the first report of a liberibacter species in the psyllid genus *Acizzia*. This new candidate liberibacter species has not been associated with plant disease, unlike other psyllid‐vectored species in the genus including ‘*Candidatus Liberibacter asiaticus*’ (CLas), ‘*Candidatus* Liberibacter africanus’ (CLaf) and ‘*Ca*. Liberibacter solanacearum’ (CLso). This study describes novel generic liberibacter genus primers, used to screen Australian psyllids for the presence of microflora that may confound diagnosis of exotic pathogens. CLbr forms a unique clade in the liberibacter genus based on phylogenetic analysis of the 16S ribosomal ribonucleic acid (rRNA) region and multilocus sequence analysis (MLSA) of seven highly conserved genes, *dnaG*,* gyrB*,* mutS*,* nusG*,* rplA*,* rpoB* and *tufB*. The MLSA mapping approach described in this article was able to discriminate between two ‘*Ca*. Liberibacter’ species within a metagenomic data set and represents a novel approach to detecting and differentiating unculturable species of liberibacter. Further, CLbr can confound the Li *et al*. (2006) quantitative PCR (qPCR) diagnostic tests for CLas and CLaf.

## Introduction

The liberibacter genus includes species of Gram‐negative alpha Proteobacteria that are known plant pathogens and endophytic species. There are currently seven species which are found in most regions of the world, excluding mainland Australia: *Liberibacter crescens* (Lcr), ‘*Candidatus* Liberibacter africanus’ (CLaf), ‘*Ca*. Liberibacter americanus’ (CLam), ‘*Ca*. Liberibacter asiaticus’ (CLas), *‘Ca*. Liberibacter caribbeanus’ (CLca), ‘*Ca*. Liberibacter solanacearum’ (CLso, synonymous to ‘*Ca*. Liberibacter psyllaurous’, CLps) and ‘*Ca*. Liberibacter europaeus’ (CLeu) (Jagoueix *et al*., 1994; Duan *et al*., 2009; Liefting *et al*., 2009a,b; Munyaneza *et al*., 2010, 2014; Raddadi *et al*., 2011; Leonard *et al*., 2012; Phahladira *et al*., 2012; Tahzima *et al*., 2014; Keremane *et al*., 2015). With the exception of Lcr, species in the liberibacter genus are associated with psyllids (Hemiptera: Psylloidea). In addition, Lcr is the only liberibacter that can be grown as an axenic culture (Leonard *et al*., 2012). Due to the fastidious nature of the predominant liberibacter species, next‐generation sequencing (NGS) approaches have played a large role in the investigation of this genus. NGS will aid the expansion of this genus and will continue to inform information on current species, their insect and plant host range.

CLso, CLas, CLam and CLaf are all exotic plant pathogens (EPPs) in Australia. In the event of an incursion, these phytopathogenic *Ca*. Liberibacter species would pose a severe threat to the Australian potato, tomato, carrot (CLso) and citrus industries (CLas, CLaf or CLam). None of the psyllid species that are known to host and vector the phytopathogenic ‘*Ca*. Liberibacter’ species are currently present in mainland Australia, yet Australia is a centre of psyllid diversity with an estimated 446 species present (Yen, 2002). Little is known about the microflora of native Australian psyllids, if the native microflora could confound diagnostic tests for phytopathogenic ‘*Ca*. Liberibacter’ species and whether native Australian psyllids could vector or host species of liberibacter. All diagnostic tests for species of liberibacter have been developed outside of Australia (Bové, 2006; Hocquellet *et al*., 1999; Jagoueix *et al*., 1994; Li *et al*., 2009; Morgan *et al*., 2012; Thompson *et al*., 2015; Wen *et al*., 2009, 2013). Therefore, Australian psyllid microflora that is not associated with plant diseases could yield false positives with the diagnostic tests, resulting in significant, unnecessary responses and economic and trade consequences. Thus, understanding the microflora of Australian psyllids is important for both biosecurity preparedness and response management.

*Acizzia solanicola,* the eggplant psyllid, is a native Australian psyllid that has broadened its host range from the native solanaceous host plant the rock nightshade, *Solanum pterophilum,* to eggplants, *Solanum melongena* (and other introduced ornamental and weed solanaceous plant species) (Taylor and Kent, 2013). Additional hosts for *A. solanicola* include wild tobacco bush, *S. mauritianum*, cape gooseberry, *Physalis peruviana*, and an undetermined species of angel's trumpet *Brugmansia* (Kent and Taylor, 2010; Taylor and Kent, 2013). *Bactericera cockerelli*, the tomato potato psyllid, is able to vector CLso, feeding on solanaceous plants including the eggplant (Liefting *et al*., 2009a,b). Due to the overlap of host plant species with a known plant pathogen vector, *A. solanicola* was chosen as a case study to investigate whether native Australian psyllids may harbour microflora that are closely related to phytopathogenic species of liberibacter. This study is the first to use generic primers to screen native Australian psyllids for liberibacter species, followed by multilocus sequence analysis (MLSA) to detect a new candidate species of liberibacter that is not associated with disease symptoms.

## Results

### Development of generic *Liberibacter* genus primers

The generic liberibacter genus primer pair, LG774F‐LG1463R, developed in this study successfully amplified the expected 684 base pair (bp) amplicons from the 16S ribosomal ribonucleic acid (rRNA) region of CLas, CLso and CLeu. When applied to DNA extracts from *A. solanicola* from Brunswick, Victoria, the LG774F‐LG1463R primer pair amplified a 684‐bp product in 17 of 37 individual psyllid DNA extracts tested. The universal 16S bacterial primer set, FD2‐RP1 (Weisburg *et al*., 1991), successfully amplified a product of approximately 1500 bp for all 37 *A. solanicola* DNA extracts.

*Acizzia solanicola* DNA extracts from six additional sites tested from Victoria (VIC) and New South Wales (NSW) in south‐eastern Australia were tested using the liberibacter generic primers, LG774F‐LG1463R and universal 16S bacterial primer set, FD2‐RP1. All 31 extracts successfully amplified the 1500‐bp product for FD2‐RP1. None of the DNA extracts from the six additional sites amplified the 684‐bp product for LG774F‐LG1463R.

Four of the 17 LG774F‐LG1463R positive PCR products from Brunswick *A. solanicola* were sequenced in both directions, and all four consensus sequences were identical to each other. Nucleotide Basic Local Alignment Search Tool (BLASTn) analysis of the longest consensus sequence (607 bp in length) determined that the LG774F‐LG1463R amplicon from *A. solanicola,* had a per cent identity of 99.0% to CLso CLso‐ZC1 (NR_074494.1 and CP002371.1), CLso Garden‐City‐KS‐5 (FJ914619.1), CLso Garden‐City‐KS‐1 (EU921626.1), CLso NZ083338 (EU935004.1), CLso TXZC018 (EU980389.1), CLps PRR1 (EU812559.1), CLps Tom100 (EU812558.1), CLso Tx15 (EU812556.1) and CLso NZ082226 (EU834130.1).

### Cloning and phylogenetic analyses of the 16S rRNA region of bacteria within *A. solanicola*

To obtain a longer region of 16S rRNA for phylogenetic analysis, the 16S rRNA FD2‐RP1 amplicons (of all bacteria amplified) from two LG774F‐LG1463R positive *A. solanicola* DNA extracts from Brunswick were cloned. Cloned colonies were screened for the presence of sequence belonging to the liberibacter genus using the LG774F‐LG1463R primer pair. Positive clones were selected for Sanger sequencing of the full‐length FD2‐RP1 amplicon. BLASTn was used to compare the longest 16S rRNA consensus sequence generated (1464 bp) (KY077741) to the GenBank non‐redundant (nr) database. The highest sequence identity was to CLaf PTSAPSY (NZ_CP004021.1) with 98.3% (100% query cover and E‐value of 0.0). This was closely followed by CLas A4 (NZ_CP010804.1) with 98.1%, CLas Ishi‐1 (NZ_AP014595.1) with 98.1%, CLas psy62 (NC_012985.3) with 98.1%, CLas gxpsy (CP04005.1) with 98.0% and CLas GuangXi‐GL‐1 (DQ778016.1) with 97.7% (all with 100% query cover and E‐value of 0.0).

**Figure 1:**
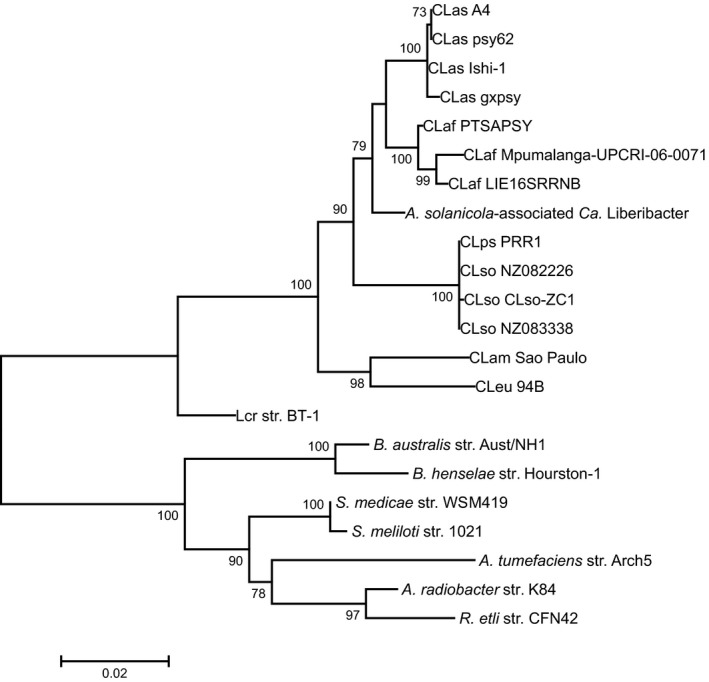
Phylogenetic analysis of the cloned 16S rRNA region of the *A. solanicola*‐associated ‘*Ca*. Liberibacter’ species. Unrooted maximum likelihood tree for the 16S rRNA region (1403 bp) was generated in MEGA6 [based on 1000 bootstraps and the Kimura 2‐parameter model with Gamma distribution and Invariable sites (K2 + *G*+*I*)]. The tree includes the cloned 16S rRNA region of the *A. solanicola*‐associated ‘*Ca*. Liberibacter’ species, all known species of the liberibacter genus and close relatives as specified in Table 2.

Phylogenetic analysis of the cloned 16S rRNA region revealed that the ‘*Ca*. Liberibacter’ species detected in *A. solanicola* is a relatively early branching, unique lineage sequence, differentiating prior to the CLas and CLaf clade (Fig. 1).

### Assembly of *‘Ca*. Liberibacter’ genes for multilocus sequence analyses

**Figure 2:**
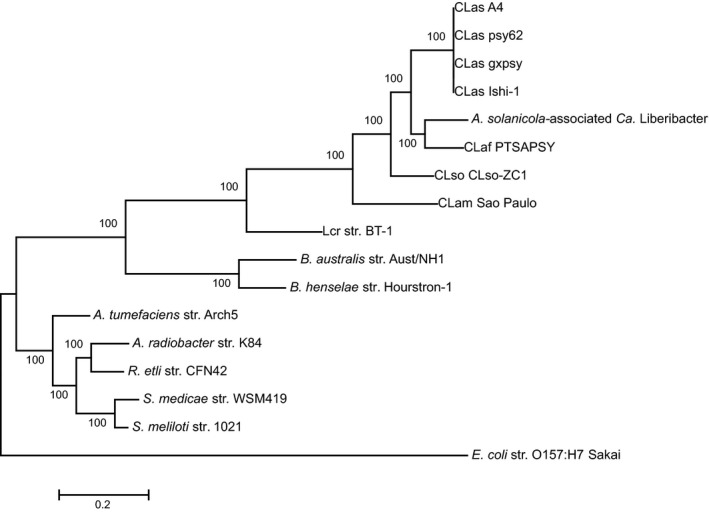
Phylogenetic analysis of the multilocus sequence of the *A. solanicola*‐associated ‘*Ca*. Liberibacter’ species. Maximum likelihood tree of seven concatenated loci, *dnaG, gyrB, mutS, nusG, rplA, rpoB* and *tufB* (a total of 12 617 bp), was generated in MEGA6 [based on 1000 bootstraps, general time reversible model with gamma distribution and invariable sites (GTR+*G*+*I*)]. The tree includes the *A. solanicola*‐associated ‘*Ca*. Liberibacter’ species, species the liberibacter genus and close relatives as specified in Table 2, this tree was rooted to *E. coli* str. O157:H7 Sakai.

The complete sequence of a further seven bacterial genes, dnaG (KY172970), gyrB (KY172971), mutS (KY172972), nusG (KY172973), rplA (KY172974), rpoB (KY172975) and tufB (KY172976), was assembled from the adapter and quality trimmed *A. solanicola* metagenome NextSeq data set (1 182 585 144 reads) (Fig. 2A). Of the assembled contigs for each gene, a single contig with full gene coverage and the highest nucleotide identity with corresponding genes from known ‘Ca. Liberibacter’ species was selected. BLASTn results revealed the highest nucleotide identity for six of the seven genes was to CLaf PTSAPSY (NZ_CP004021.1); dnaG (84.7%), gyrB (87.1%), mutS (84.7%), nusG (85.1%), rpoB (87.7%) and tufB (85.1%). The highest nucleotide identity for rplA (87.1%) was to CLas A4 (NZ_CP010804.1).

To validate the assembly of the seven selected genes from the *A. solanicola* metagenome NextSeq data set, simulated reads of CLaf PTSAPSY (NZ_CP004021.1) genome were run through the bioinformatics workflow (Fig. 2B). All seven genes were successfully re‐assembled for the simulated reads of CLaf PTSAPSY (NZ_CP004021.1) with a nucleotide identity of 100.0% to CLaf PTSAPSY (NZ_CP004021.1). When the simulated reads were spiked into the *A. solanicola* metagenome NextSeq data set (Fig. 2C), multiple contigs were assembled for each of the seven genes. The BLASTn searches confirmed the presence of assembled contigs for each gene for both the *A. solanicola*‐associated ‘*Ca*. Liberibacter’ species and for CLaf PTSAPSY (NZ_CP004021.1) and clearly differentiated between the two species, with identical nucleotide sequence identity to the individual analyses.

### Multilocus sequence analysis and phylogenetic analyses for the *A. solanicola*‐associated ‘*Ca*. Liberibacter’ species

No recombination events were recognized in Recombination Detection Program 4.56 (RPD4) (Martin *et al*., 2015) for the DNA sequences of the seven genes incorporated into the MLSA for the liberibacter genus analysed.

A distance matrix of the concatenated contigs representing all seven genes covering 12 379 bp determined that the *A. solanicola*‐associated ‘*Ca*. Liberibacter’ species has 86.1%, 84.1%, 82.2%, 75.7% and 69.5% sequence identity to CLaf, CLas, CLso, CLam and Lcr respectively (Table 1). The inclusion of four CLas genomes in the distance matrix showed high sequence identity within a species, with a sequence identity of 100% for the seven concatenated genes between the CLas isolates (Table 1, highlighted in bright blue).

**Table 1:**
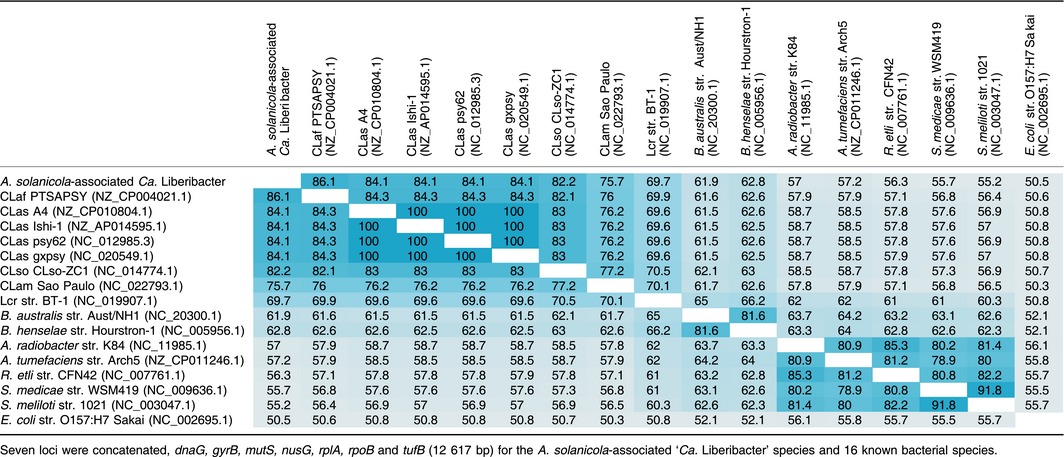
Distance matrix of the concatenated MLSA sequence data

Phylogenetic analysis of the concatenated genes differentiated the *A. solanicola*‐associated ‘*Ca*. Liberibacter’ species into a separate clade within the liberibacter genus and was most closely related to CLaf PTSAPSY (NZ_CP004021.1) (Fig. 2). The concatenated phylogenetic tree presented the same trend in topology as the phylogenetic trees for individual genes, with the *A. solanicola*‐associated ‘*Ca*. Liberibacter’ species consistently forming a single clade between CLaf and CLas (Fig. S1A–G). All genes had bootstrap support (≥ 97%) for the separation of the *A. solanicola*‐associated ‘*Ca*. Liberibacter’ species with the exception of *nusG* and *rplA* which produced poor bootstrap support for the separation of CLaf and the *A. solanicola*‐associated ‘*Ca*. Liberibacter’ species (59% for *nusG* and 32% for *rplA*). However, *nusG* also showed poor bootstrap support for the separation of CLas and CLaf (69%) (Fig. S1D). The bootstrap support significantly increased for the concatenated phylogeny (Fig. 2), showing very strong bootstrap support (100%) for the separation of all clades of bacteria that were analysed.

### Diagnostic quantitative PCR (qPCR) analyses of *A. solanicola*‐associated ‘*Ca*. Liberibacter’ species

A positive qPCR result was generated from the *A. solanicola*‐associated ‘*Ca*. Liberibacter’ DNA extracts using the HLBas and HLBaf detection protocol (Li *et al*., 2006). A negative qPCR result was generated from *A. solanicola*‐associated ‘*Ca*. Liberibacter’ DNA using the HLBam (Li *et al*., 2006) and LsoF (Li *et al*., 2009) detection protocols. Negative results were generated for all *A. solanicola* DNA extracts that were negative for the *A. solanicola*‐associated ‘*Ca*. Liberibacter’ species.

The nucleotide sequence alignment of the pathogenic diagnostic forward primers and the 16S rRNA target region on the *A. solanicola*‐associated ‘*Ca*. Liberibacter’ species reveal six and nine identical base pairs at the 3′ end binding site for HLBas and HLBaf respectively (Fig. S2). The reverse primer and probe for these detection protocols are identical.

## Discussion

A new candidate liberibacter species was detected in the native Australian eggplant psyllid, *A. solanicola*, based on the sequence and phylogenetic analyses of the 16S rRNA region and confirmed with MLSA of seven concatenated genes. The proposed name for this *A. solanicola*‐associated species is ‘*Candidatus* Liberibacter brunswickensis’ (CLbr), after Brunswick, a suburb in Melbourne, Victoria, from where the psyllid host was first collected. CLbr was not associated with plant disease, instead the discovery arose during the screening of Australian native psyllids with newly developed generic liberibacter genus primers.

The generic liberibacter genus primers amplified CLas, CLeu and CLso *in vitro* and 14 sequences representing all known non‐pathogenic and phytopathogenic species of liberibacter with sequence data available *in silico,* while excluding closely related bacterial genera (*in silico*). When used with DNA extracted from *A. solanicola,* a single amplicon of the expected size was generated from DNA extracts from 17 of 37 individual psyllids. Direct sequencing of these LG774F‐LG1463R amplicons determined that the sequence was most closely related to CLso sequences (99% sequence identity), but was not an identical match. The high sequence identity of this amplicon to CLso highlights the risk of falsely identifying a liberibacter species on regions of the 16S rRNA, as is done in amplicon metagenomics. No additional *A. solanicola* DNA extracts from six sites across south‐east Australia produced an amplicon using the liberibacter generic primer pair. These sites were selected based on collection sites previously reported (Kent and Taylor, 2010; Taylor and Kent, 2013). Kent and Taylor (2010) report damage to eggplants caused by *A. solanicola* feeding; however, the microflora of these psyllids was not tested and this may be due to the volume of psyllids feeding.

This study demonstrates that the generic liberibacter primers are a powerful tool to screen hosts for species of liberibacter. To confirm the phylogenetic position of the *A. solanicola*‐associated ‘*Ca*. Liberibacter’ within the liberibacter genus, a larger region of the 16S rRNA was sequenced and the MLSA was conducted. Phylogenetic analysis of the 16S rRNA region separated CLbr (KY077741) into a unique clade, which branched relatively early and prior to the CLas and CLaf clades (Fig. 1). Significant branch bootstrap support (100%) was obtained with phylogenetic analysis of the concatenated genes (Fig. 2) confirming that CLbr separated into a unique clade and that this new species is most closely related to, but distinct from, CLaf PTSAPSY (NZ_CP004021.1). The phylogeny for each individual gene displayed the same topology as the concatenated tree for CLbr (Fig. S1A–G), however bootstrap values varied, supporting the need for the concatenated MLSA mapping approach.

Previous MLSA for unculturable bacteria, including the majority of species within the liberibacter genus, has been limited to cloning and chromosome walking techniques (Planet *et al*., 1995; Teixeira *et al*., 2008; Lin *et al*., 2011). An increase in the availability of next‐generation sequencing techniques has allowed a small number of full or near complete (< 10 contigs) genomes of CLas (Duan *et al*., 2009; Lin *et al*., 2013; Katoh *et al*., 2014; Zheng *et al*., 2014; Wu *et al*., 2015), CLaf (Lin *et al*., 2015), CLam (Wulff *et al*., 2014) and CLso (Lin *et al*., 2011; Thompson *et al*., 2015) to be assembled. To date for the candidate liberibacter species, there are seven complete liberibacter genomes and an extra five near complete genomes publically available. Although this is a small number, these genomes have allowed comparisons to Lcr which can be grown in axenic culture and have provided insights to the fastidious nature of the liberibacter genus (Leonard *et al*., 2012; Fagen *et al*., 2014; Wulff *et al*., 2014). However, it is common for genome sequences in the liberibacter genus to have multiple long repetitive regions and multiple variable prophage inserts, making assembly of a complete genome challenging, even with a reference genome present (Thompson *et al*., 2015; Wu *et al*., 2015).

**Figure 3:**
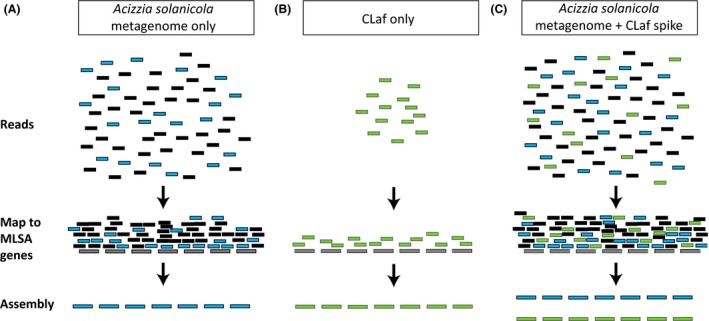
Graphical representation of the MLSA approach of raw reads being mapped to seven reference genes and the assembly of these reads for the NextSeq *A. solanicola* metagenome data set (A), CLaf only (B) and CLaf spiked NextSeq *A. solanicola* metagenome data set (C). The *A. solanicola*‐associated ‘*Ca*. Liberibacter’ species and CLaf species are highlighted in blue and green respectively. All other reads in the metagenome data set are shaded black, and the reference genome CLas is shaded grey.

Large complex data sets, such as the *A. solanicola* metagenome sequenced in this study, also present significant challenges to genome assembly. The MLSA mapping approach using known liberibacter reference genes to extract reads for assembly, enabled MLSA to be completed on an undescribed organism within a metagenomic data set. This approach was further validated by spiking the *A. solanicola* metagenomic data set with simulated reads from the complete genome of CLaf PTSAPSY (NZ_CP004021.1) followed by reassembly of the seven genes used for the MLSA analysis for both ‘*Ca*. Liberibacter’ species. The CLaf PTSAPSY genome was selected for validation as it is phylogenetically the closest species to CLbr and therefore the most likely to generate chimeric contigs during assembly. This method was able to differentiate a novel species of bacteria in the liberibacter genus and also provide assurance that multi‐species sequence chimeras had not formed. Individual data sets for both CLbr (metagenomics data from *A. solanicola*, Fig. 3A) and CLaf PTSAPSY (NZ_CP004021.1) (simulated reads, Fig. 3B) assembled into identical contigs to those assembled from the spiked data set (Fig. 3C). This PCR‐independent approach has the potential to identify multiple liberibacter species present in the one host, allows species identification without the need to obtain an axenic culture of bacteria and is a useful analytical tool for whole genome shotgun sequence data sets that contain obligate non‐culturable organisms. The seven genes selected in this study are highly conserved within the liberibacter genus and were able to differentiate the novel ‘*Ca*. Liberibacter’ from known species. However, the seven genes are not suitable to separate haplotypes within a liberibacter species (Table 1 and Fig. 2). To apply the MLSA mapping approach to other unculturable bacterial genera, it is recommended to identify genes that are highly conserved within the genus.

The phylogenetic relationship of CLbr to CLaf and CLas may support the proposed theory of the Gondwana supercontinent origins of CLaf and CLas, as Australia was once a part of Gondwana before dislocation approximately 160 million years ago (Teixeira *et al*., 2008; Nelson *et al*., 2013; Bové, 2014). In addition, the *Acizzia* genus is distributed from Australia, New Zealand, the Old World tropics and extending to North Africa, the Middle East and the Mediterranean (references within Taylor and Kent, 2013). This may infer that liberibacter species have been present within, and evolved with psyllids over time, although as a secondary symbiont this is likely to be a more recent event than that of the evolution of the primary symbiont, ‘*Ca*. Carsonella’ (Brumin *et al*., 2012; Sloan and Moran, 2012). Further research on the biology of the bacterium within its psyllid host is needed to determine the multiple co‐speciation events. To date, *A. solanicola* has been detected in Australia, and most recently New Zealand (Taylor and Kent, 2013). Based on sequence analyses, species within the liberibacter genus do not group according to plant species in which they are disease causing or endophytic. This is shown with CLam, pathogenic to citrus, grouping closer to CLeu and Lcr endophytes in pear and papaya (Raddadi *et al*., 2011; Leonard *et al*., 2012), supporting the theory that these bacteria may have evolved with their insect hosts.

This is the first time a liberibacter species has been reported in mainland Australia. Without plant disease symptoms present or reported, this discovery arose due to the need to conduct surveillance for liberibacter species of biosecurity significance to Australian agriculture. CLbr gave a false‐positive result for the diagnostic qPCR methods reported in (Li *et al*., 2006) for CLas and CLaf but not for CLso and CLam (Li *et al*., 2006, 2009). This shows that there is microflora in native Australian psyllids that is able to confound diagnostic tests developed outside of Australia. Australia is a centre for psyllid diversity, and *A. solanicola* was the first psyllid species to be screened with the generic liberibacter genus primers. The detection of CLbr is an indication that the number of species that have been identified in the liberibacter genus is a huge underrepresentation and the genus will increase beyond the current eight recorded species. Further work into the biology, host range and distribution of CLbr is underway.

## Experimental procedures

### Insect collection and morphological identification

A colony of *A. solanicola* was collected from an eggplant, *Solanum melongena* Black Beauty in Brunswick (a metropolitan suburb of Melbourne), Victoria (VIC), Australia. No disease symptoms were seen on the eggplant, nor on the cape gooseberry, *Physalis peruviana* (cultivar unknown) on which an additional colony of *A. solanicola* was also established at the collection site. The eggplant and psyllid colony was transferred to the laboratory and kept in insect cages (BugDorm, Taiwan) at 20°C (± 2°C) and 60% (± 5%) relative humidity. Individuals from the psyllid colony were morphologically confirmed as *A. solanicola* (AgriBio, 5 Ring Road, Victoria 3083, Australia).

An additional 31 *A. solanicola* individuals were collected from *S. melongena* or *S. mauritianum* across six sites in south‐eastern Australia. These sites include Bundoora (VIC, nine psyllids), Kew (VIC, two psyllids), Bellingen (NSW, nine psyllids), Clybucca (NSW, five psyllids), Dorrigo West (NSW, three psyllids) and Karangi (NSW, three psyllids).

### DNA extraction

DNA was extracted using the Qiagen DNeasy Blood and Tissue Kit (Qiagen, Germany) with the following amendments. Individual psyllids were placed into a 1.5‐mL tube (Eppendorf, Germany) containing two 3 mm glass beads and 20 μl of proteinase K (Qiagen, Germany). Insects were homogenized using a Bead‐Mill TissueLyser (Qiagen, Germany) at 30 megahertz (Mhz) for 1 min and were centrifuged at 17 000× gravitational force (g) for 20 s (s). The slurry was re‐homogenized at 30 Mhz for 1 min, centrifuged a second time for 20 s, 180 μl of ATL buffer (Qiagen, Germany) added and the protocol completed according to manufacturer's instructions (Qiagen, Germany) with a final elution volume of 100 μl.

To allow an increased concentration of DNA to be extracted from an individual psyllid for Illumina sequencing, the cetyl trimethyl ammonium bromide (CTAB) method (Murray and Thompson, 1980) was used with the following amendments. Individual psyllids were placed into a 1.5‐mL tube (Eppendorf, Germany) containing two 3 mm glass beads, 50 μl of CTAB buffer (2% CTAB, 1.4 M NaCl, 1% PVP‐40, 0.02 M EDTA and 0.1 M Tris‐HCl, pH 8.0) and insects were homogenized as described above, using a Bead‐Mill TissueLyser (Qiagen, Germany). After precipitation, DNA was resuspended in 15 μl of UltraPure distilled water (Invitrogen, Netherlands).

DNA quantity and quality was estimated from each extraction using the Nanodrop ND‐1000 Spectophotometer (Thermo Scientific, Germany) and Qubit 2.0 fluorometer (Invitrogen, Germany) and then stored at −20°C.

### Development of generic *Liberibacter* genus primers

To identify microflora in Australian psyllids that is phylogenetically close to the liberibacter genus, generic liberibacter genus‐specific primers were designed to amplify sequences from known liberibacter species encompassing both non‐pathogenic and pathogenic species, while excluding phylogenetically related bacterial genera. CLca was not able to be included in this analysis due to insufficient sequence data available (currently 1125 bp of the 16S rRNA region for both GenBank additions: KP012550.1 and KP012551.1). Full‐length nucleotide sequences of the 16S rRNA region of the remaining six liberibacter species and species of phylogenetically related alpha Proteobacteria (Table 2 – Sequence alignment of the 16S rRNA region) were aligned using MUSCLE (Edgar, 2004) in MEGA6 (Tamura *et al*., 2013). PCR primers were designed using Primer3plus (http://primer3plus.com/) to target regions identified in the alignment that were conserved between liberibacter species, while excluding phylogenetically related alpha Proteobacteria.

**Table 2:** Bacteria used for sequence alignments and phylogenetic analyses

Bacterium species and strain/isolate	Accession number	Sequence alignment and phylogenetic analysis		Class: Order
		16S rRNA region	MLSA	
CLaf PTSAPSY	NZ_CP004021.1	●	●	Alpha Proteobacteria: Rhizobiales
CLam Sao Paulo	NC_022793.1	●	●	Alpha Proteobacteria: Rhizobiales
CLas gxpsy	NC_020549.1	●	●	Alpha Proteobacteria: Rhizobiales
CLas Ishi‐1	NZ_AP014595.1	●	●	Alpha Proteobacteria: Rhizobiales
CLas A4	NZ_CP010804.1	●	●	Alpha Proteobacteria: Rhizobiales
CLas psy62	NC_012985.3	●	●	Alpha Proteobacteria: Rhizobiales
CLso CLso‐ZC1	NC_014774.1	●	●	Alpha Proteobacteria: Rhizobiales
Lcr str. BT‐1	NC_019907.1	●	●	Alpha Proteobacteria: Rhizobiales
*Bartonella henselae* str. Hourstron‐1	NC_005956.1	●	●	Alpha Proteobacteria: Rhizobiales
*Bartonella australis* str. Aust/NH1	NC_020300.1	●	●	Alpha Proteobacteria: Rhizobiales
*Agrobacterium tumefaciens* str. Arch5	NZ_CP011246.1	●	●	Alpha Proteobacteria: Rhizobiales
*Agrobacterium radiobacter* str. K84	NC_011985.1	●	●	Alpha Proteobacteria: Rhizobiales
*Sinorhizobium meliloti* str. 1021	NC_003047.1	●	●	Alpha Proteobacteria: Rhizobiales
*Sinorhizobium medicae* str. WSM419	NC_009636.1	●	●	Alpha Proteobacteria: Rhizobiales
*Rhizobium* etli str. CFN42	NC_007761.1	●	●	Alpha Proteobacteria: Rhizobiales
CLaf Mpumalanga‐UPCRI‐06‐0071	EU754741.1	●		Alpha Proteobacteria: Rhizobiales
CLaf str. LEI16SRRNB	L22533.1	●		Alpha Proteobacteria: Rhizobiales
CLso str. NZ083338	EU935004.1	●		Alpha Proteobacteria: Rhizobiales
CLso str. NZ082226	EU834130.1	●		Alpha Proteobacteria: Rhizobiales
CLps PRR1	EU812559.1	●		Alpha Proteobacteria: Rhizobiales
CLeu 94B	JX244260.1	●		Alpha Proteobacteria: Rhizobiales
*Escherichia coli* O157:H7 str. Sakai	NC_002695.1		●	Gamma Proteobacteria: Enterobacteriales

Forward and reverse primers, LG774F (5′‐GTAAACGATGAGTGCTAGCTGTTGGG‐3′) and LG1463R (5′‐CTGACCRTACCGTGGCCGG‐3′), were selected to produce an amplicon of 684 bp and tested against DNA extracts of CLas, CLeu and CLso. DNA extractions of 37 individual *A. solanicola* psyllids isolated from the colony at different times were tested using these liberibacter generic primers. Reactions were performed in 25 μl containing 1 μl of DNA template, 10× PCR buffer (Invitrogen, Netherlands), 1.5 mM MgCl_2_, 0.2 mM dNTPs, 0.25 μM of each primer and 1 U of Platinum Taq polymerase (Invitrogen, Netherlands). The PCR conditions were denaturation at 95°C for 3 mins, then 35 cycles of 95°C for 30 s, 62°C for 30 s and 72°C for 1 min, plus a final step at 72°C for 5 mins. A second PCR using the primer pair FD2 (5′‐CCGAATTCGTCGACAACAGAGTTTGATCATGGCTCAG‐3′) and RP1 (5′‐CCCGGGATCCAAGCTTACGGTTACCTTGTTACGACTT‐3′) was performed (Weisburg *et al*., 1991) to amplify the 16S rRNA region of all bacterial species present within the *A. solanicola* samples to ensure that DNA extraction had been successful. Amplification was performed in an Eppendorf Mastercycler pro PCR System (Fisher Scientific, United States of America), and PCR products were visualized by agarose gel electrophoresis (1.5% agarose gel) with SYBR Safe DNA gel stain (Invitrogen, Netherlands).

Amplicons were purified using the Wizard SV gel and PCR Clean‐Up System (Promega, United States of America) according to manufacturer's instructions. Sequence of the purified PCR products was generated via capillary Sanger sequencing in both directions, performed by the Australian Genome Research Facility (AGRF), Melbourne, Australia. Consensus sequences were generated using Geneious 7.0 (Kearse *et al*., 2012) and used as queries against the GenBank nr database using BLASTn version 2.4.0 (Altschul *et al*., 1990) for homologous sequences, available at the National Centre for Biotechnology Information (NCBI).

### Cloning and sequencing of the 16S rRNA region of liberibacter*‐*like bacteria within *A. solanicola*

The FD2‐RP1 product of the 16S rRNA region (approximately 1500 bp) was cloned from *A. solanicola* DNA extracts that tested positive using the generic liberibacter genus primers LG774F‐LG1463R. The amplicon generated using the generic bacterial primers for 16S rRNA region amplification, FD2‐RP1, was cloned into the pGEM –T Easy vector (Promega, United States of America) following manufacturer's instructions. To obtain colonies with inserts closely related to the 16S rRNA region of liberibacter species, DNA from isolated colonies was screened by PCR using the liberibacter generic primers LG774F‐LG1463R. Plasmid DNA from LG774F‐LG1463R positive FD2‐RP1 colonies was purified by centrifugation using the PureLink Quick Plasmid Miniprep Kit (Invitrogen, Netherlands) according to manufacturer's instructions and sequenced via capillary Sanger sequencing (AGRF, Melbourne, Vic., Australia). Consensus sequences were analysed as described above.

### Library preparation and Illumina sequencing of *A. solanicola*

Illumina sequencing was performed on a single DNA extract of *A. solanicola* that tested positive using the liberibacter generic, LG774F‐LG1463R, primer pair. The quality and quantity of the *A. solanicola* DNA was estimated using the Nanodrop ND‐1000 Spectophotometer (Thermo Scientific) and double‐stranded DNA (dsDNA) HS assay for the Qubit 2.0 fluorometer (Invitrogen). DNA was diluted to 0.2 ng μl^−1^, and a library was prepared using the Nextera XT DNA Library Preparation kit (Illumina) following the manufacturer's instructions. Library quality was checked using the Qubit 2.0 fluorometer (Invitrogen, Netherlands), KAPA Library Quantification Kit (KAPA Biosystems, United States of America), and the 2200 TapeStation system (Agilent, United States of America) according to the High Sensitivity D1000 protocol. Initial paired‐end sequencing (2 × 250 bp) was performed on an Illumina MiSeq (Reagent Kit v3) to establish library quality, optimal library concentration and estimate the required sequencing depth. A final paired‐end sequencing run (2 x 150 bp) was performed on an Illumina NextSeq (Reagent Kit v2).

### *In silico* multilocus sequence analysis (MLSA)

Seven highly conserved genes were selected for MLSA of the unidentified ‘*Ca*. Liberibacter’. These included DNA primase (*dnaG*), DNA mismatch repair protein (*mutS*) (Glynn *et al*., 2012), DNA gyrase subunit B (*gyrB*) (Marrero *et al*., 2013; Mee *et al*., 2015), Transcription termination/antitermination protein NusG (*nusG*), 50S ribosomal protein L1 (*rplA*), DNA‐directed RNA polymerase subunit beta *(rpoB*) and a single copy of Elongation factor Tu (*tufB*) (Teixeira *et al*., 2008).

Sequence reads from the NextSeq *A. solanicola* metagenome data set were adapter and quality trimmed using TrimGalore (Krueger, 2012), and mapped to the seven genes listed above from the complete genome of CLas gxpsy (NC_020549.1) using BWA‐MEM (Li, 2013) with relaxed parameters for a mismatch penalty (reduced from 4 to 2). Reads that mapped were assembled using SPAdes *de novo* version 3.6.0 (Bankevich *et al*., 2012) with kmer lengths of 107, 87, 71 and 31 (Fig. 3A). Resulting contigs were compared to known sequences in GenBank using the BLASTn algorithm (Altschul *et al*., 1990) to identify the ‘*Ca*. Liberibacter’ sequences and to confirm the gene identity.

To establish that one species of bacteria can be identified from a large metagenomic sequence data set using the above MLSA mapping approach, and that the resulting contigs are not merely a chimera of bacterial reads within the sample, one million simulated sequence reads (150 bp in length) were generated using wgsim version 0.3.0 (Li, 2011) from the most closely related species to the *A. solanicola*‐associated ‘*Ca*. Liberibacter’ (based on 16S rRNA phylogenetic analysis), CLaf PTSAPSY (NZ_CP004021.1). The simulated reads were mapped directly to the seven reference genes of CLas gxpsy (NC_020549.1) to ensure that the genes re‐assembled from the simulated reads (Fig. 3B). The simulated reads were then spiked into the NextSeq *A. solanicola* metagenome data set, and all reads were mapped to the seven reference loci listed above (Fig. 3C). For both mapping exercises, the MLSA extraction, assembly and identification were repeated as described above.

### Phylogenetic analysis

Cloned 16S rRNA region sequence, the assembled contigs of *dnaG*,* gyrB*,* mutS*,* nusG*,* rplA*,* rpoB* and *tufB* of the new ‘*Ca*. Liberibacter’ from *A. solanicola* and the concatenated multilocus sequence were aligned with sequences of the liberibacter genus and sequences of closely related bacteria using MUSCLE (Edgar, 2004) according to Table 1. The concatenated liberibacter genus sequences were screened for presence of recombination with Recombination Detection Program 4.56 (RDP4) (Martin *et al*., 2015) using the default parameters for RDP, GENECONV, Chimaera, MaxChi, BOOTSCAN and SISCAN methods.

Phylogenetic analyses were performed for the 16S rRNA region, the seven individual genes (Fig. S1) and the concatenated MLSA using the program MEGA6 (Tamura *et al*., 2013) employing the maximum likelihood method. A bootstrap consensus tree was inferred using 1000 samplings of the data, and bootstrap values below 70% were not shown. For each alignment, 24 different substitution models were compared using the Find Best DNA Model Test in MEGA6. The best substitution model was selected by the lowest BIC score (Bayesian Information Criterion) and applied to each phylogenetic analyses accordingly (Kimura, 1980; Lanave *et al*., 1984; Nei and Kumar, 2000; Zwickl and Holder, 2004). Nearest‐Neighbor‐Interchange (NNI) algorithm (Nei and Kumar, 2000), Neighbor Joining/Bio Neighbor Joining (Saitou and Nei, 1987) and very strong branch swap filter settings were used to obtain the initial tree. *Escherichia coli* O157:H7 str. Sakai was used as an out‐group and a root placed on this branch for the MLSA phylogenetic analyses (including individual gene trees, supporting information). In addition, a distance matrix of the concatenated genes was generated.

### Diagnostic qPCR analyses of *A. solanicola*‐associated ‘*Ca*. Liberibacter’ species

Two *A. solanicola* DNA extracts that were positive for the *A. solanicola*‐associated ‘*Ca*. Liberibacter’ species and two *A. solanicola* DNA extracts that were negative for the *A. solanicola*‐associated ‘*Ca*. Liberibacter’ species were tested using diagnostic qPCR protocols for CLas, CLaf, CLam and CLso (Li *et al*., 2006, 2009) according to authors instructions using the QuantStudio 3 Real‐Time PCR System and the QuantiTect Multiplex PCR NoROX Kit (Qiagen, Germany). To assess primer binding regions, the phytopathogenic ‘*Ca*. Liberibacter’ species‐specific primers and probe were aligned to the target 16S rRNA region of *A. solanicola*‐associated ‘*Ca*. Liberibacter’ using Mega6.

## Supporting information


**Fig. S1.** Phylogenetic analysis for each gene in the MLSA.

**Fig. S2.** Sequence alignment for the 16S RNA region for the primer binding regions of the phytopathogenic ‘*Ca*. Liberibacter’ species‐specific forward primers (HLBas, HLBaf, HLBam and LsoF) to the *A. solanicola*‐associated ‘*Ca*. Liberibacter’ species.
