# Supplementary figures and images for: Novel ‘Candidatus Liberibacter’ species identified in the Australian eggplant psyllid, Acizzia solanicola
Source: Microb Biotechnol. 2017 Apr 7;10(4):833–44. doi: 10.1111/1751-7915.12707 (PMC5481521; doi:10.1111/1751-7915.12707)

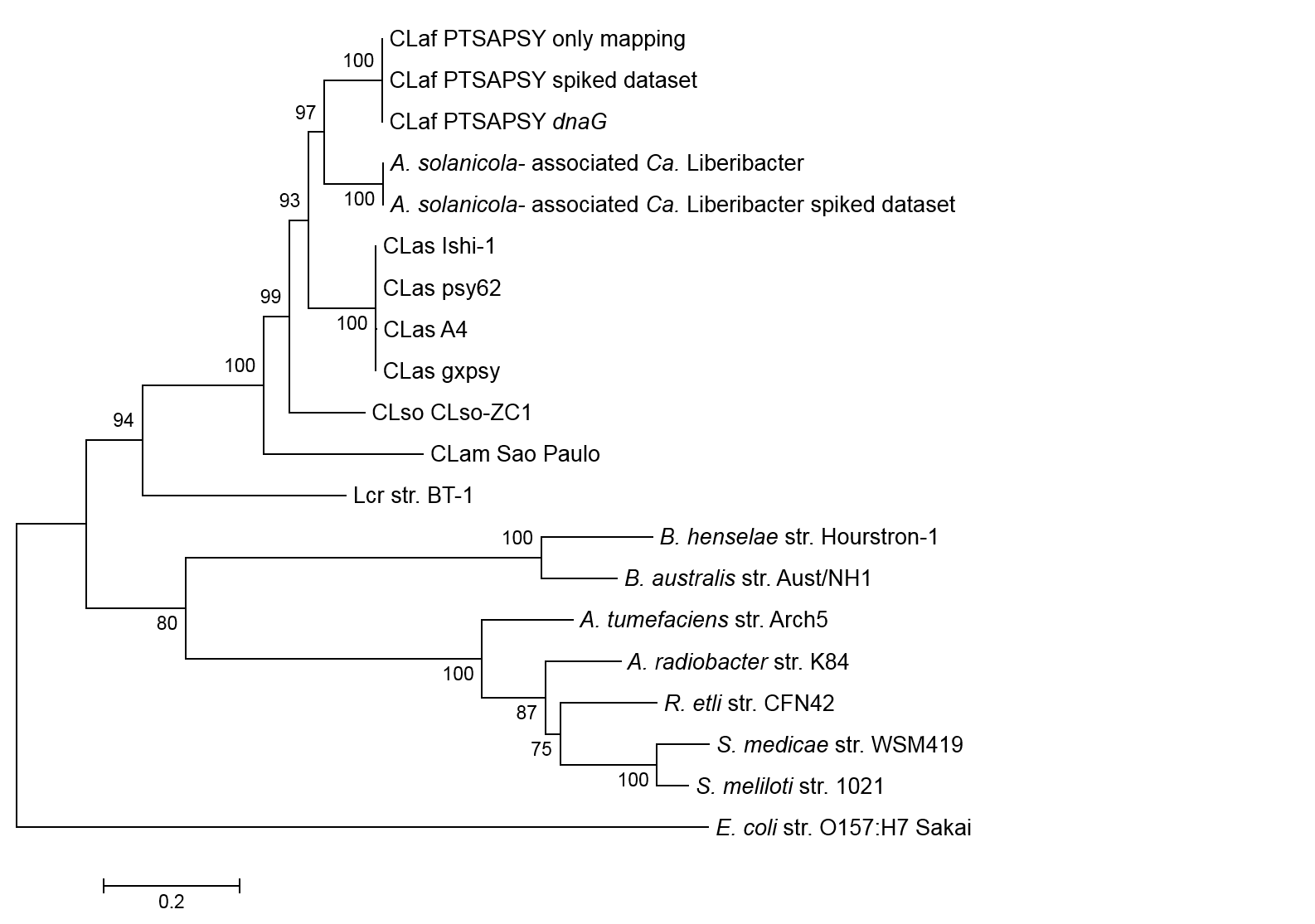

Supplement: Supplementary file 1 — Fig. S1. Phylogenetic analysis for each gene in the MLSA. [file MBT2-10-833-s001.tif]

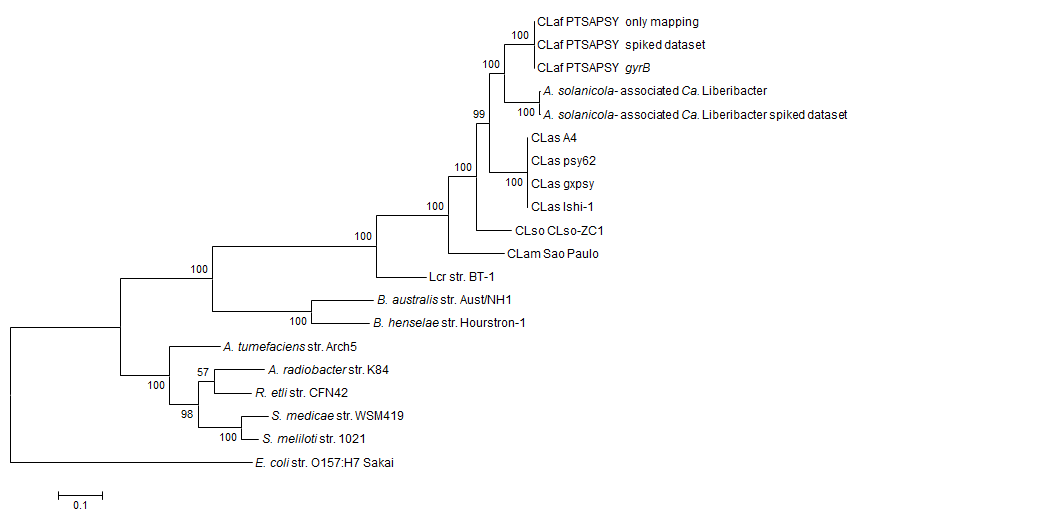

Supplement: Supplementary file 2 [file MBT2-10-833-s002.tif]

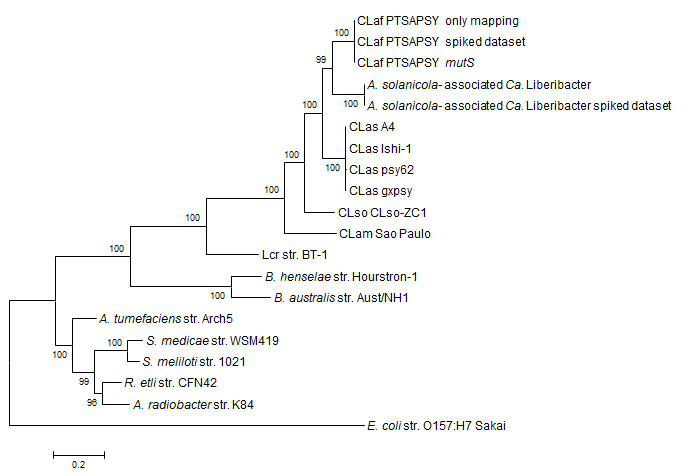

Supplement: Supplementary file 3 [file MBT2-10-833-s003.tif]

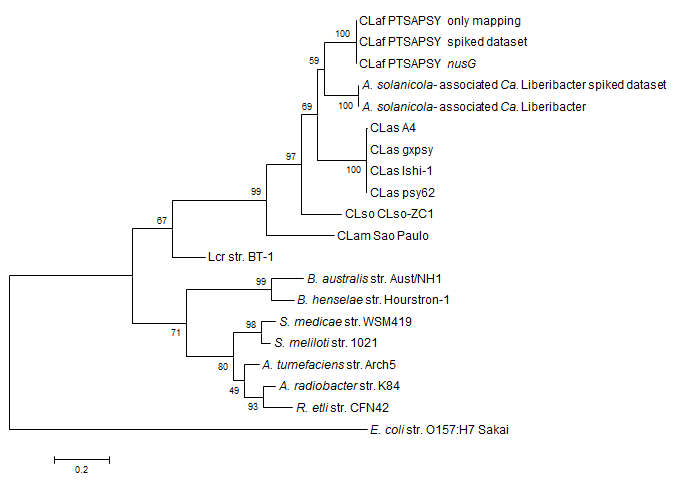

Supplement: Supplementary file 4 [file MBT2-10-833-s004.tif]

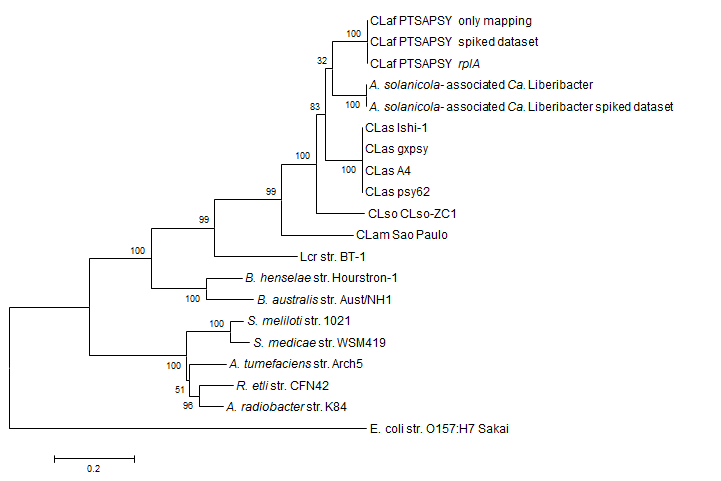

Supplement: Supplementary file 5 [file MBT2-10-833-s005.tif]

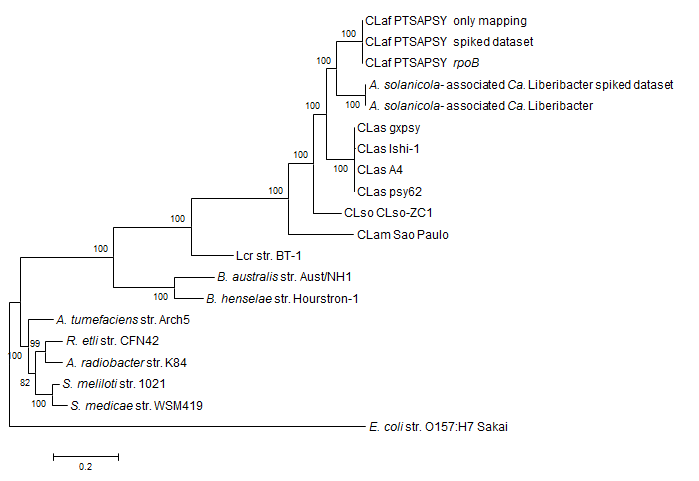

Supplement: Supplementary file 6 [file MBT2-10-833-s006.tif]

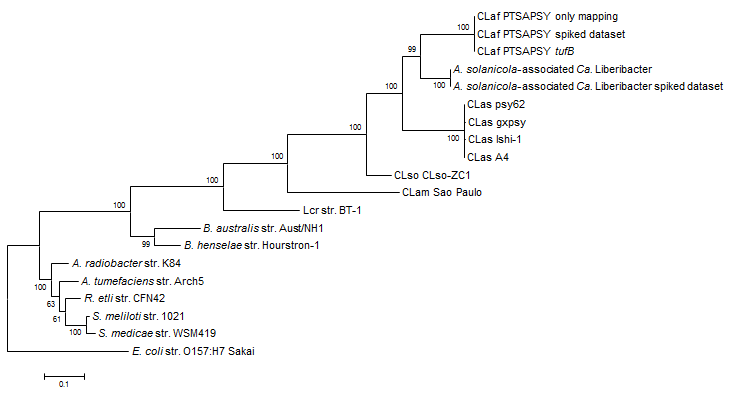

Supplement: Supplementary file 7 [file MBT2-10-833-s007.tif]

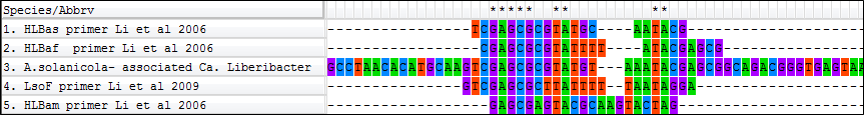

Supplement: Supplementary file 8 — Fig. S2. Sequence alignment for the 16S RNA region for the primer binding regions of the phytopathogenic ‘Ca. Liberibacter’ species‐specific forward primers (HLBas, HLBaf, HLBam and LsoF) to the A. solanicola‐associated ‘Ca. Liberibacter’ species. [file MBT2-10-833-s008.tif]
